# Supplementary material for: Mechanism of the Conformational Change of the Protein Methyltransferase SMYD3: A Molecular Dynamics Simulation Study
Source: Int J Mol Sci. 2021 Jul 2;22(13):7185. doi: 10.3390/ijms22137185 (PMC8267938; doi:10.3390/ijms22137185)
Supplement: Supplementary file 1 [file ijms-22-07185-s001.zip › ijms-1280228-supplementary.pdf]

Mechanism of the conformational change of the protein methyltransferase SMYD3: a molecular dynamics simulation study

Jixue Sun[1,#], Zibin Li[1,#] and Na Yang[1,*]

1. State Key Laboratory of Medicinal Chemical Biology, College of Pharmacy and Key Laboratory of Medical Data Analysis and Statistical Research of Tianjin, Nankai University, 300353 Tianjin, China

* To whom correspondence should be addressed. Email: yangnanku@nankai.edu.cn

[#] These authors contribute equally to this work

SI Materials and Methods

SAXS measurements and analyses

SMYD3 protein was expressed and purified as previously reported[1,2]. Four groups of protein and protein complexes, including Apo, SAM, MAP3K2 and SAM_MAP3K2 were prepared for SAXS measurements. Each group contains three samples at different protein concentrations of 1.0 mg/mL, 2.5 mg/mL, and 5.0 mg/mL, respectively. Cofactors including MAP3K2 peptide (final concentration 5 mM) or/and SAM (1 mM) were incubated on ice with SMYD3 for 1 h before SAXS measurement. SAXS data were collected at beamline BL19U2 of National Center for Protein Science Shanghai Synchrotron Radiation Facility (SSRF). The wavelength of X-ray radiation was set at 1.033 Å. Scattered X-ray intensities were collected using a Pilatus 1 M detector (DECTRIS Ltd,). The sample-to-detector distance was set such that the detecting range of momentum transfer ($q = 4\pi\sin\theta/\lambda$, where 2θ is the scattering angle) of SAXS experiments was 0.01 to 0.30 $Å^{-1}$. To reduce the radiation damage, a flow cell made of cylindrical quartz capillary was used. SAXS data were collected at 10 °C using 60 μL sample incubation buffer (20 mM Tris-HCl pH 8.0, 100 mM NaCl, 5% glycerol) as 20 × 1 s exposures. Scattering profiles for 20 exposures were compared to exclude data showing radiation damage and averaged. Radial frame averaging and buffer subtraction were done using standard protocols using the software package BioXTAS RAW. Linear guinier plots in Guinier region ($q*Rg < 1.3$) were confirmed and the RoG were evaluated using Guinier's approximation in all experimental groups. Fitting of the experiment curves and the theoretical curves were computed from models were performed using CRYSOL.

Hamiltonian function

The potential was calculated using the Hamiltonian function[3]

$$U = V_{bonded} + V_{nobonded}$$
$$= V_b(r_{ij}) + V_a(\theta_{ijk}) + V_d(\varphi_{ijkl}) + V_{LJ}(r_{ij}) + V_c(r_{ij})$$
$$= \frac{1}{2} k_{ij}^{r} (r_{ij} - r_0)^2 + \frac{1}{2} k_{ijk}^{\theta} \left[\theta_{ijk} - \theta_0\right]^2 + k_{ijkl}^{\varphi}\left[1 + \cos(n\varphi_{ijkl} - \varphi_0)\right] +$$
$$4\epsilon_{ij}\left[\left(\frac{\sigma_{ij}}{r_{ij}}\right)^{12} - \left(\frac{\sigma_{ij}}{r_{ij}}\right)^{6}\right] + \frac{q_i q_j}{4\pi\epsilon_0\varepsilon_r r_{ij}} \qquad (1),$$

The potential includes bonded term (bond, angle and dihedral energies) and non-bonded term (Van der Waals and electrostatic energies). In the bonded term, i, j, k and l represent atoms i, j, k and l, r_{ij} and r_0 represent the current and equilibrium bond length, respectively. The same applies to the angle and dihedral energies. k_{ij}^{r}, k_{ijk}^{θ} and k_{ijkl}^{φ} were elastic coefficients for bond, angle and dihedral energies determined by various of atoms, respectively. In the non-bonded term, ϵ_{ij} represents well depth, σ_{ij} represents Van der Waals radius, q represents atom charge, ϵ_0 and ε_r represent dielectric constant in vacuum and medium, respectively. These parameters are supported in the AMBER ff14SB force field[4].

Reference:

1 W. Fu, N. Liu, Q. Qiao, M. Wang, J. Min, B. Zhu, R. M. Xu and N. Yang, Structural basis for substrate preference of SMYD3, a SET domain-containing protein lysine methyltransferase, *J. Biol. Chem.*, 2016, **291**, 9173–9180.

2 J. Sun, F. Shi and N. Yang, Exploration of the Substrate Preference of Lysine Methyltransferase SMYD3 by Molecular Dynamics Simulations, *ACS Omega*, 2019, **4**, 19573–19581.

3 W. D. Cornell, P. Cieplak, C. I. Bayly, I. R. Gould, K. M. Merz, D. M. Ferguson, D. C. Spellmeyer, T. Fox, J. W. Caldwell and P. a. Kollman, A second generation force field for the simulation of proteins, nucleic acids, and organic molecules, *J. Am. Chem. Soc.*, 1995, **117**, 5179–5197.

4 J. A. Maier, C. Martinez, K. Kasavajhala, L. Wickstrom, K. E. Hauser and C. Simmerling, ff14SB: Improving the Accuracy of Protein Side Chain and Backbone Parameters from ff99SB, *J. Chem. Theory Comput.*, 2015, **11**, 3696–3713.

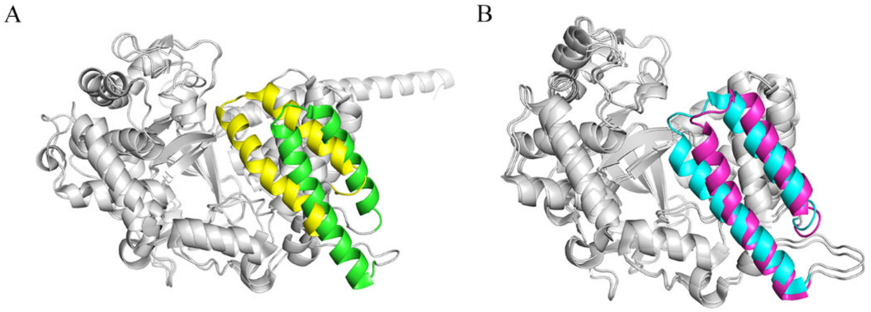

Figure S1. Superpositions of the crystal structure of SMYD family. (A) The open CTD conformation of SMYD1 (PDB ID: 3N71), colored in green and the closed CTD conformation of SMYD3 (PDB ID: 5EX0), colored in yellow. Only the two helixes in the CTD are colored for comparison purposes. (B) The two different CTD conformation of SMYD2 with the cofactor SAH and the inhibitor sinefungin (PDB ID: 3QWV and 3QWW), colored in cyan and magenta, respectively.

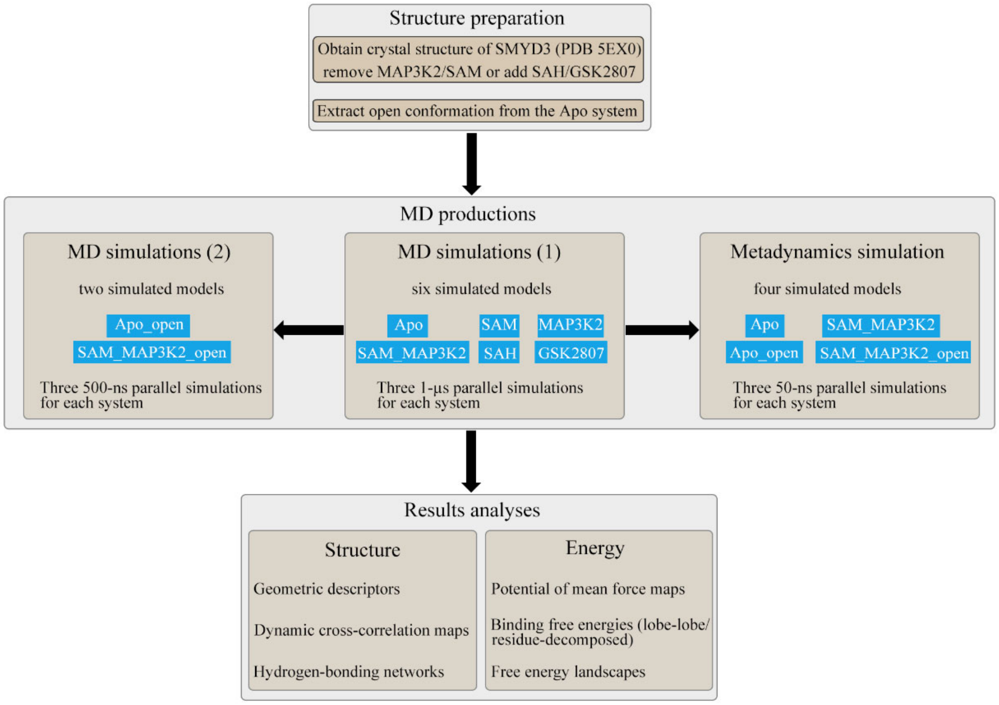

Figure S2. Molecular simulation protocol.

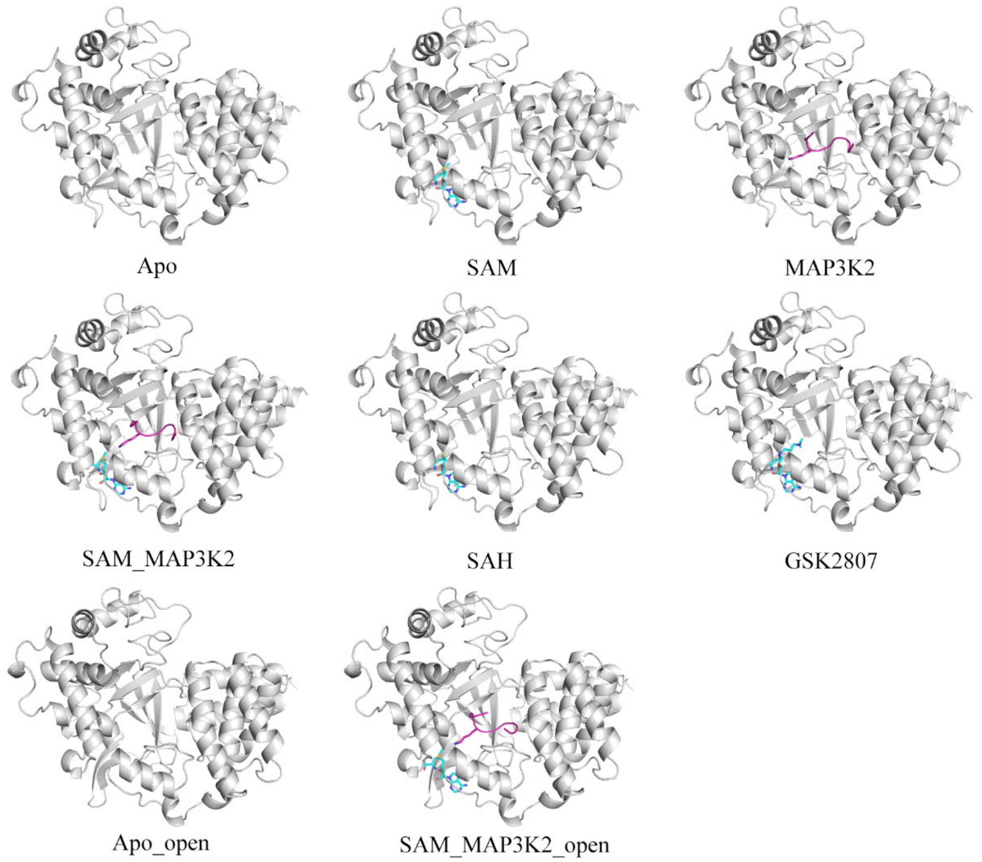

Figure S3. The initial structure for each simulated model. SAM/SAH/GSK2807 and the MAP3K2 peptide are shown in cyan and magenta, respectively.

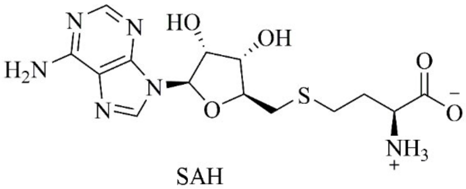

SAH

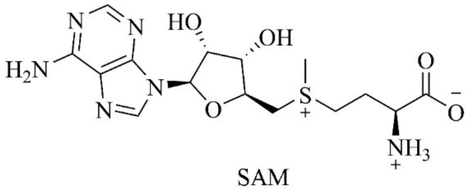

SAM

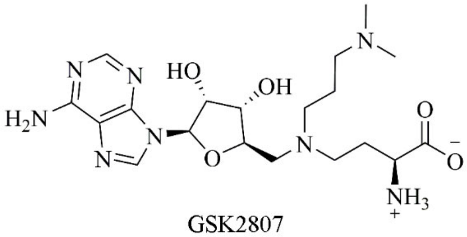

GSK2807

Figure S4. Chemical structure of SAH, SAM and GSK2807.

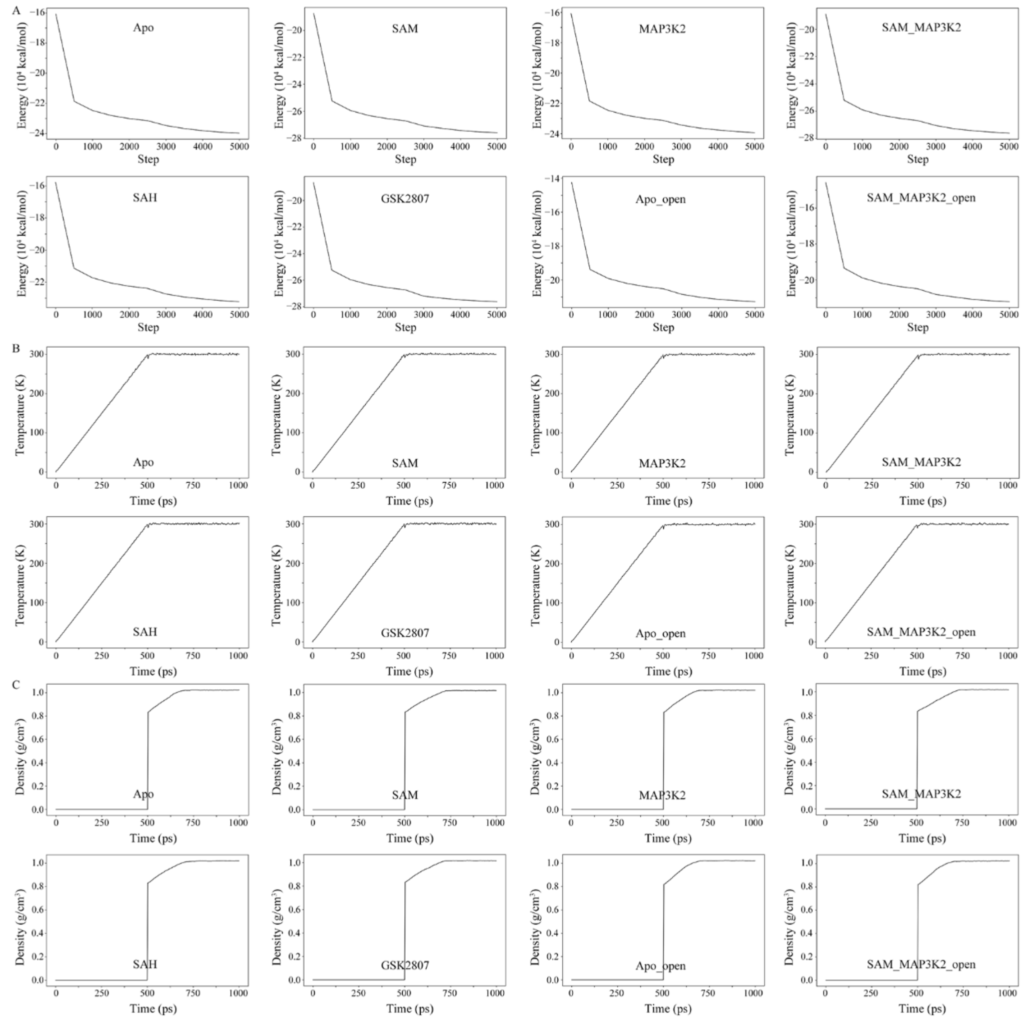

Figure S5. (A) The energy of each simulated system during minimization. (B) The temperature of each simulated system during equilibration stage including a 500-ps heating and a 500-ps density equilibration. (C) The density of each simulated system during equilibration stage.

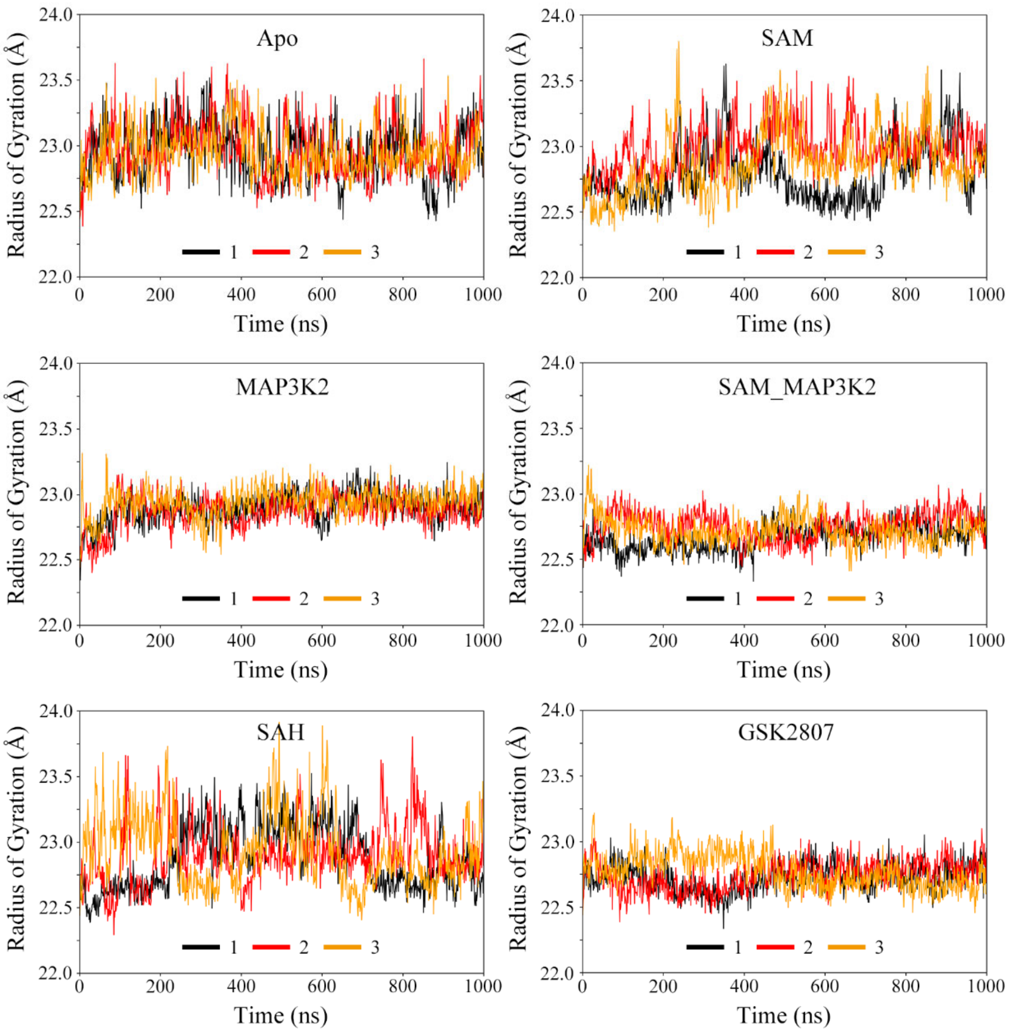

Figure S6. Time evolutions of RoG during three parallel MD simulations (black, parallel simulation 1; parallel simulation 2; orange, parallel simulation 3). The same scheme is used in the following figures unless explicitly specified.

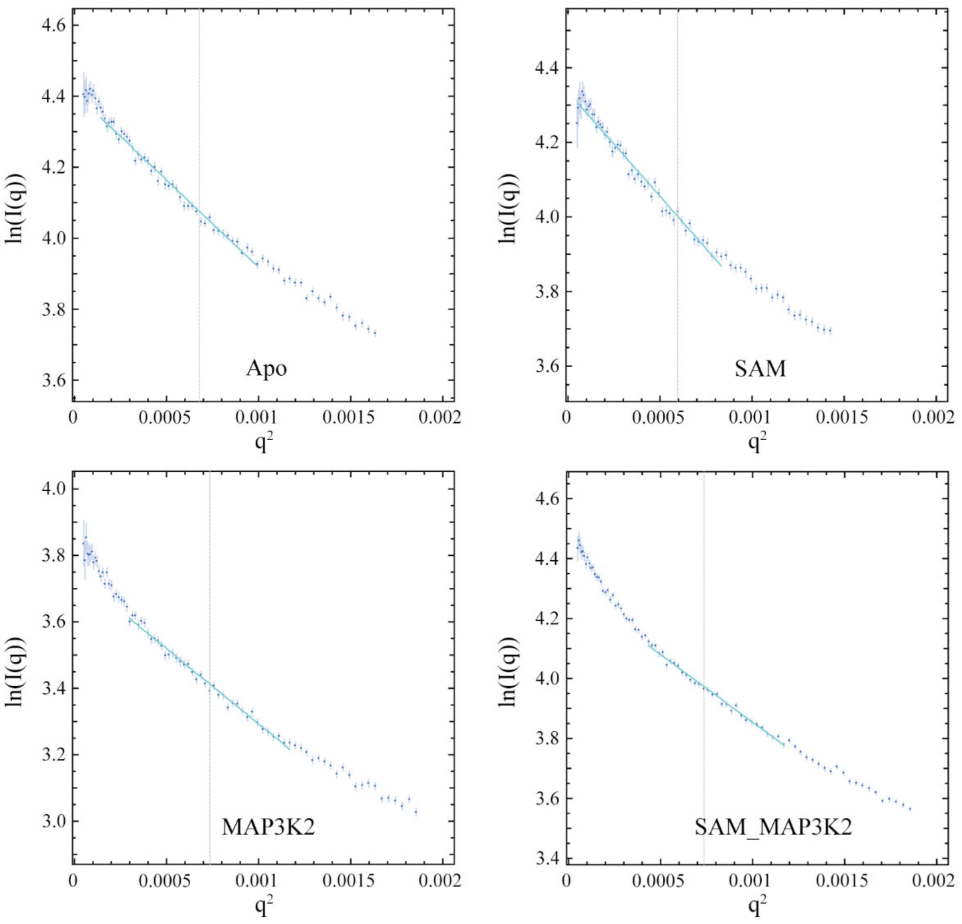

Figure S7. Guinier plots in the low-q region of four experimental groups. A series of concentrations of scattering curves from each experimental group were merged to reduce interparticle interactions influence.

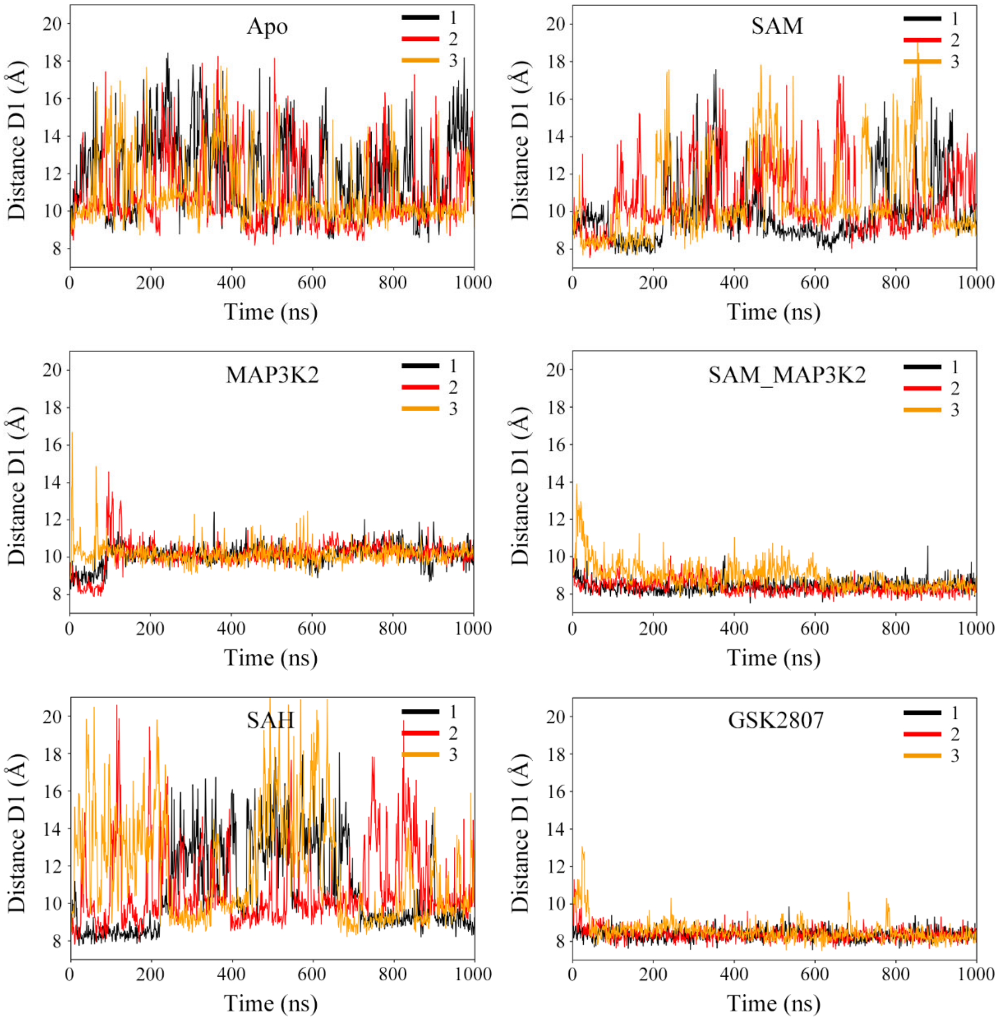

Figure S8. Time evolutions of distance D1 during three parallel MD simulations in each system.

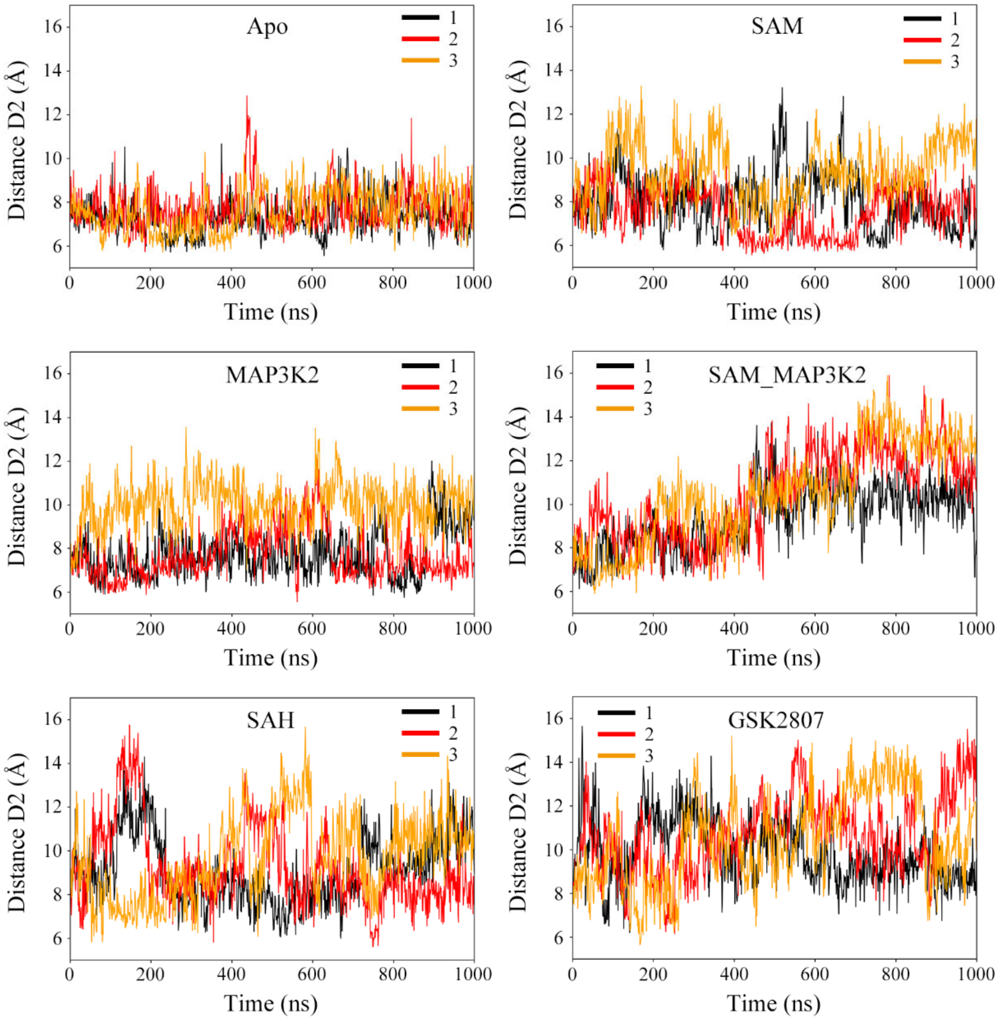

Figure S9. Time evolutions of distance D2 during three parallel MD simulations in each system.

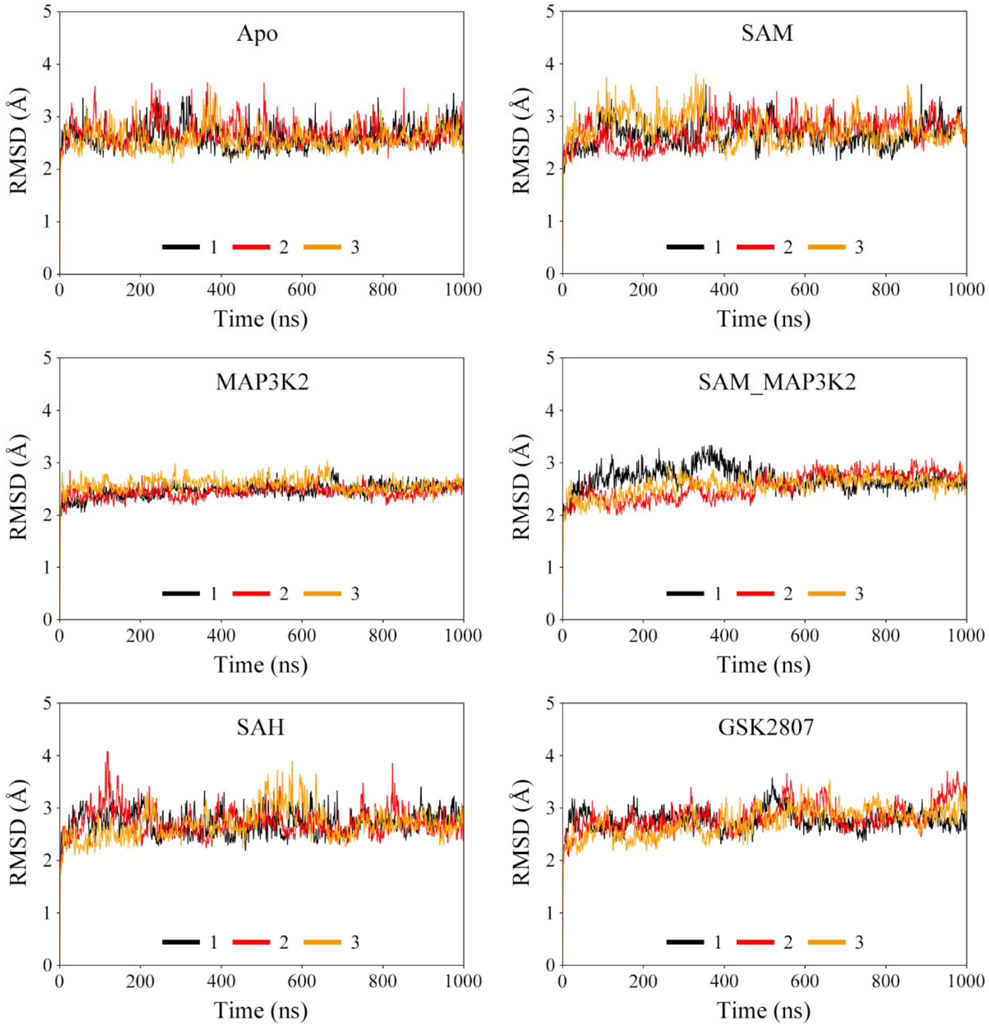

Figure S10. Time evolutions of RMSD during three parallel MD simulations in each system.

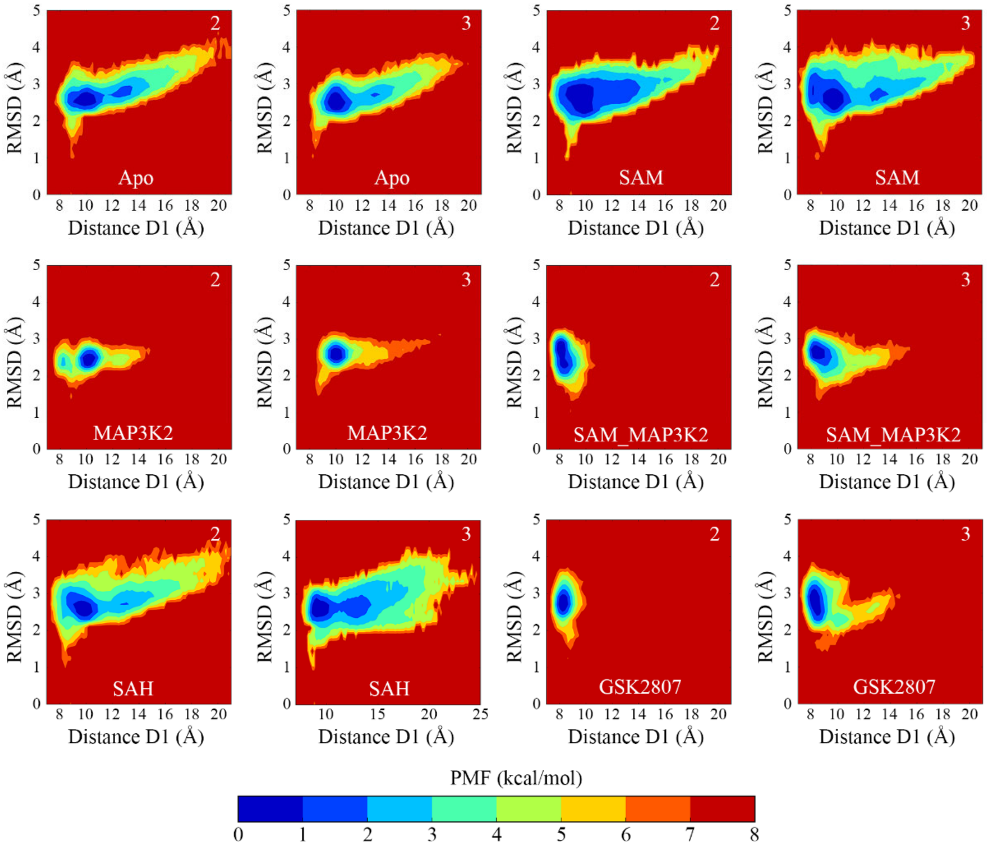

Figure S11. PMF calculated for the distance D1 vs. the RMSD of SMYD3 during the second and third parallel MD simulations in each system.

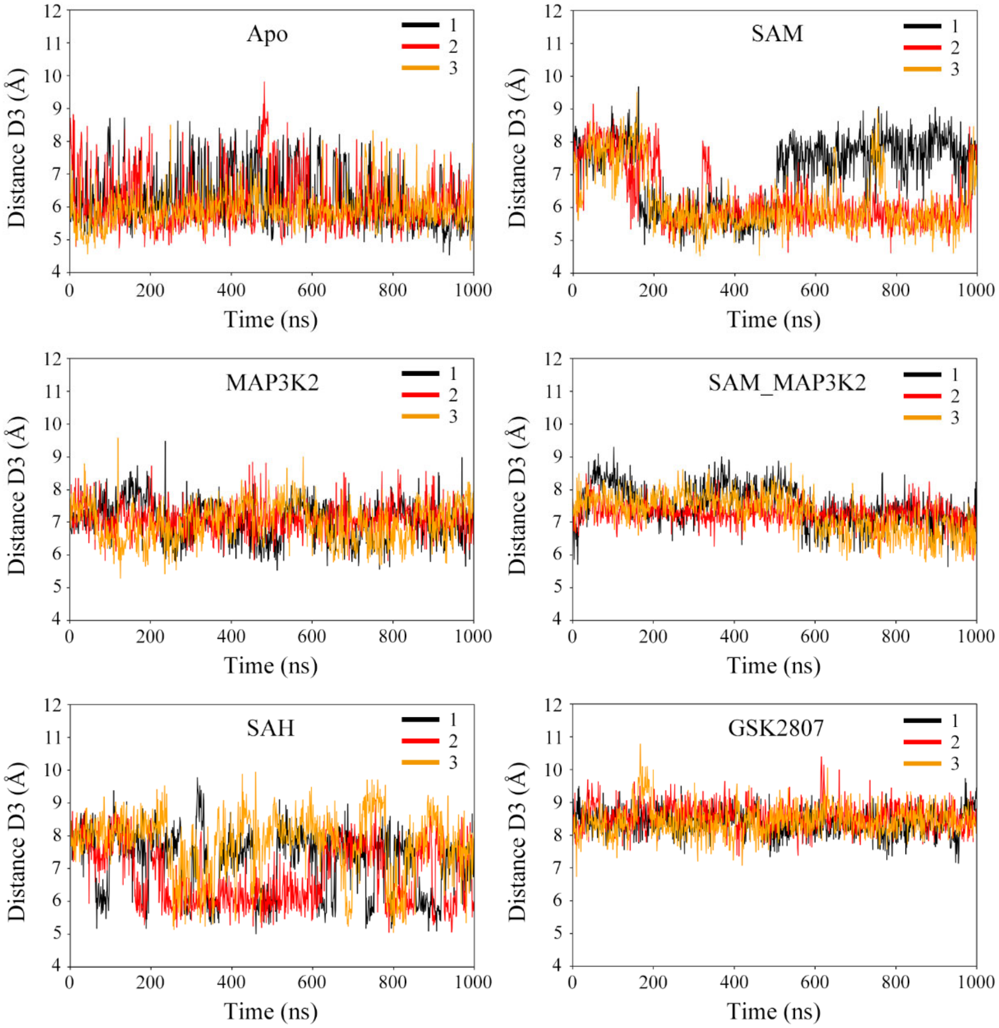

Figure S12. Time evolutions of distance D3 during three parallel MD simulations in each system.

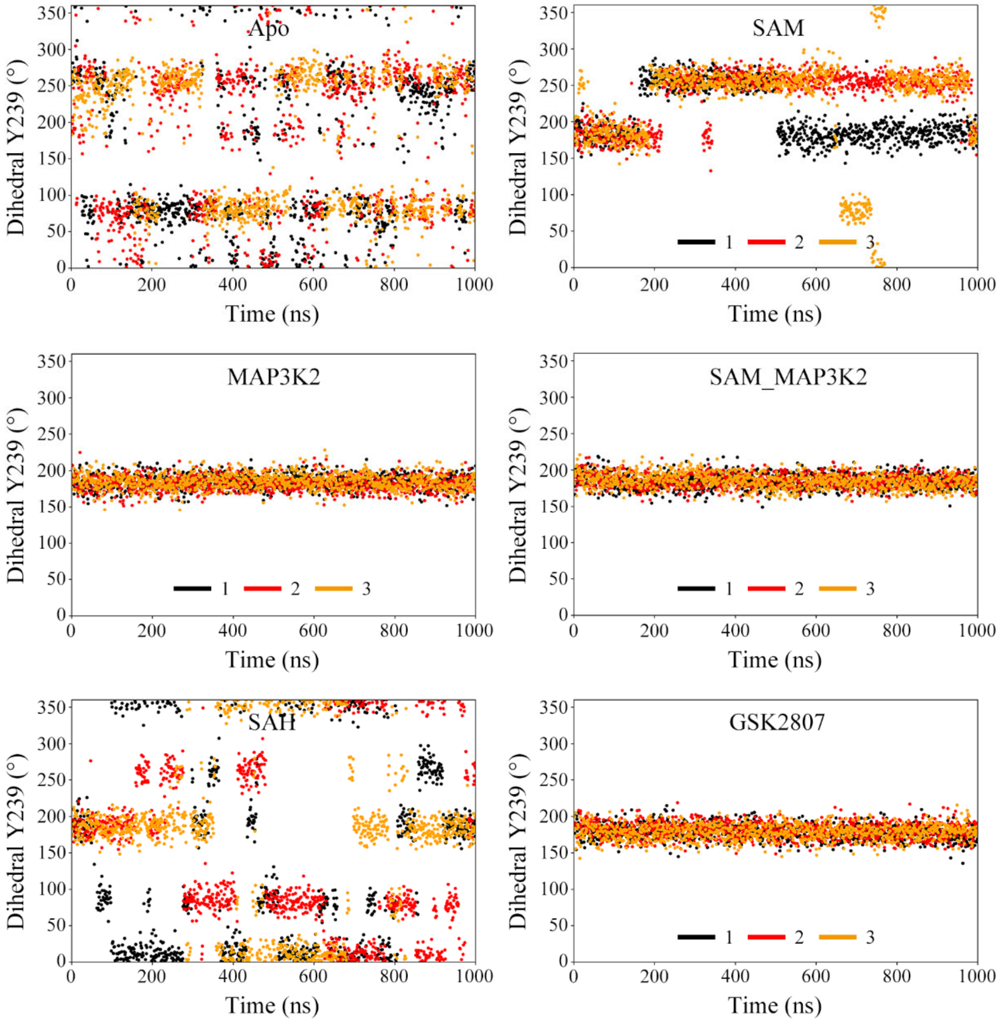

Figure S13. Time evolutions of dihedral Y239 during three parallel MD simulations in each system.

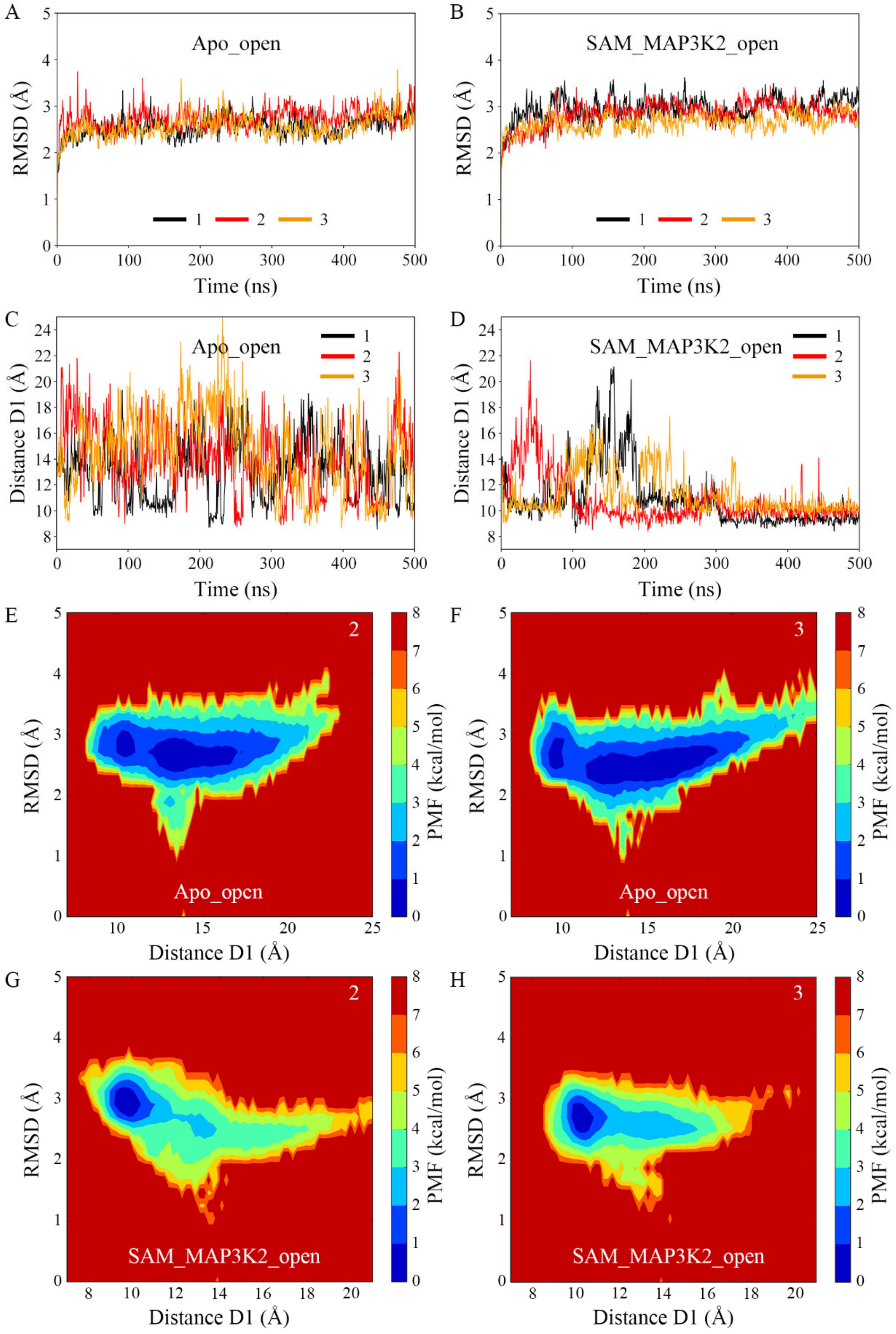

Figure S14. Time evolutions of RMSD during three parallel MD simulations in the (A) Apo_open and (B) SAM_MAP3K2_open systems. Time evolutions of distance D1 during three parallel MD simulations in the (A) Apo_open and (B)

SAM_MAP3K2_open systems. (E-H) PMF calculated for the distance D1 vs. the RMSD of SMYD3 during the second and third parallel MD simulations in each system.

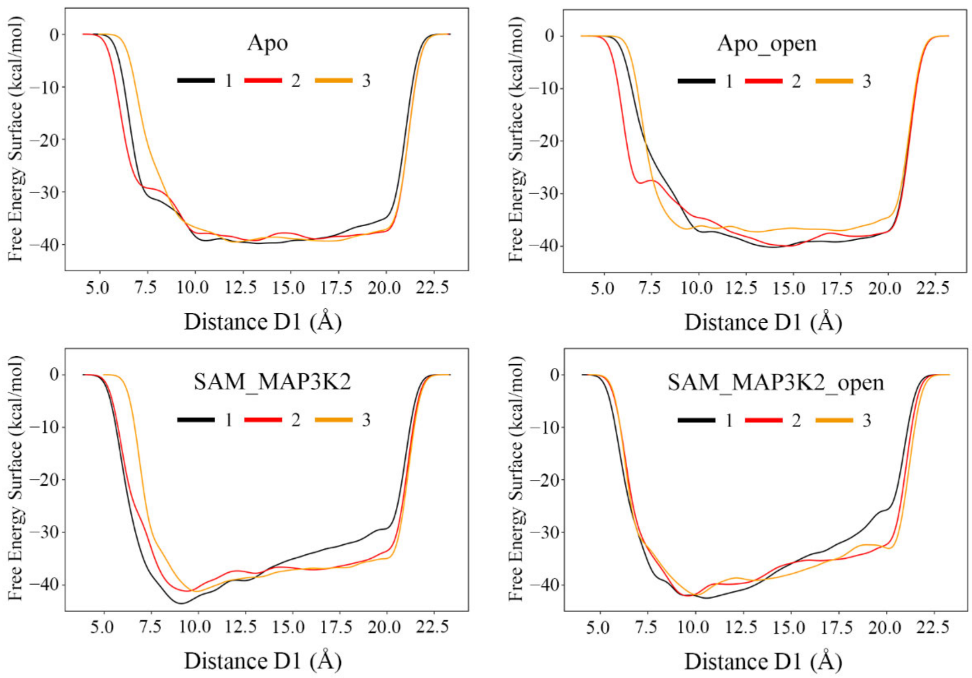

Figure S15. Free energy surface associated with the conformational change of SMYD3 as a function of the distance D1 in each of three parallel metadynamics simulations for the (A) Apo, (B) SAM_MAP3K2, (C) Apo_open and (D) SAM_MAP3K2_open systems.

Table S1. Averaged values and standard deviations of RMSD, RoG, and defined geometric descriptors during three parallel MD simulations in each system.

	Apo	SAM	MAP3K2	SAM_MAP3K2	SAH	GSK2807
RMSD	2.61±0.23[b]	2.64±0.24	2.55±0.19	2.69±0.21	2.72±0.22	2.77±0.19
	2.68±0.24	2.71±0.27	2.44±0.14	2.55±0.26	2.71±0.27	2.83±0.27
	2.58±0.23	2.75±0.29	2.58±0.15	2.72±0.24	2.68±0.29	2.74±0.27
RoG	22.95±0.20	22.79±0.21	22.89±0.11	22.66±0.10	22.87±0.24	22.72±0.11
	22.97±0.20	22.98±0.17	22.87±0.12	22.75±0.11	22.90±0.22	22.74±0.11
	22.96±0.16	22.87±0.23	22.94±0.11	22.72±0.11	22.93±0.26	22.77±0.13
D1	12.07±2.09	9.88±1.65	10.11±0.55	8.43±0.32	10.71±2.29	8.38±0.29
	10.98±1.86	10.63±1.74	10.14±0.72	8.37±0.36	10.75±2.05	8.56±0.35
	10.94±1.68	10.60±2.12	10.16±0.54	8.93±0.73	11.73±2.76	8.56±0.60
D2	7.29±0.76	8.02±1.26	7.74±1.07	9.39±1.47	9.09±1.52	9.85±1.44
	7.68±0.89	7.56±1.10	7.69±1.11	10.42±2.01	9.25±1.84	10.66±1.74
	7.62±0.96	9.36±1.34	10.00±1.05	10.47±2.24	9.62±1.87	10.44±2.05
D3	6.20±0.79	7.03±1.07	7.07±0.61	7.50±0.60	7.21±0.95	8.56±0.39
	6.14±0.83	6.25±0.90	7.11±0.46	7.27±0.34	6.79±0.94	8.39±0.40
	5.94±0.53	6.18±0.94	6.99±0.60	7.26±0.59	7.74±0.94	8.43±0.45
Dihedral Y239	152.06±99.67	208.13±37.41	185.05±9.87	184.04±10.19	144.08±120.29	180.43±10.71
	177.77±96.89	236.97±34.99	184.62±9.64	184.09±10.11	143.62±102.13	180.91±10.55
	173.69±90.19	225.77±62.42	182.08±9.72	185.33±8.96	166.48±99.71	178.34±10.64

RMSD, RoG, distance D1, D2 and D3 are in Å. Dihedral Y239 is in °.

Table S2. The statistics of RoG values of SAXS experimental groups.

Concentration	Apo	SAM	MAP3K2	SAM_MAP3K2
1.0 mg/mL	32.48	32.65	30.67	30.78
2.5 mg/mL	37.05	37.45	32.45	31.98
5.0 mg/mL	38.41	39.45	36.21	36.86

All RoG values are in Å.

Table S3. Populations of conformational states.

Conformational State	Apo	SAM	MAP3K2	SAM_MAP3K2	SAH	GSK2807
Closed Conformation	0	46.78%	0	100%	49.35%	100%
Intermediate Conformation	53.63%	40.29%	100%	0	16.75%	0
Open Conformation	46.37%	12.93%	0	0	33.90%	0

Table S4. Occupancies of hydrogen bonds at the P1-P5 positions during three parallel MD simulations in each system.

Position	Hydrogen Bond	Occupancy					
		Apo	SAM	MAP3K2	SAM_MAP3K2	SAH	GSK2807
P1	R14-D262	1.76%	71.57%	5.58%	90.83%	93.61%	86.75%
		23.99%	92.06%	5.64%	87.18%	95.74%	79.16%
		36.92%	76.07%	20.94%	67.91%	80.92%	75.54%
P2	M242-R265	13.70%	66.65%	33.55%	48.38%	49.51%	61.28%
		37.04%	55.91%	39.57%	51.82%	48.31%	61.91%
		39.96%	47.52%	47.07%	55.25%	50.41%	56.65%
P3	A188-H404	63.17%	22.38%	52.33%	3.43%	31.34%	4.11%
		55.63%	57.36%	56.45%	15.07%	41.35%	0.11%
		58.83%	32.30%	57.94%	19.47%	21.87%	4.16%
P4	S44-V193	21.49%	53.94%	16.79%	87.23%	47.21%	82.77%
		22.72%	72.71%	16.58%	81.82%	69.17%	93.42%
		45.44%	34.37%	20.64%	75.65%	26.10%	91.65%
P5	K42-E295	5.73%	17.00%	37.63%	50.51%	11.47%	43.45%
		18.84%	22.90%	55.04%	66.96%	28.27%	45.56%
		29.61%	20.34%	49.44%	43.51%	7.79%	42.68%

Table S5. Decomposed binding free energies of the SET and MYND domain toward the CTD.

ΔG	Apo	SAM	MAP3K2	SAM_MAP3K2	SAH	GSK2807
K42	0.29±0.33	0.16±0.81	−1.55±1.51	−3.11±1.61	−0.28±0.69	−2.45±1.65
G43	−0.02±0.37	−0.40±0.56	−1.10±0.55	−2.22±0.53	−0.67±0.51	−2.23±0.53
S44	−0.01±0.28	−0.15±0.25	−0.36±0.23	−1.91±0.34	−0.10±0.18	−1.91±0.38
E295	0.27±0.24	0.35±0.34	−0.16±0.78	−0.94±1.00	0.31±0.24	−0.56±1.09
H299	0.16±0.10	0.02±0.45	−0.10±0.47	−0.91±0.63	−0.08±0.30	−1.01±0.66
W300	−0.68±1.06	−2.19±1.46	−2.50±1.08	−5.75±0.82	−1.39±0.52	−6.58±1.33
P363	−1.61±0.42	−1.35±0.64	0.01±0.04	0.01±0.10	−0.63±0.28	−0.42±0.49
H404	−3.82±0.65	−2.74±1.07	−3.31±0.67	−1.31±0.99	−0.75±0.46	−1.42±1.00

All decomposed binding free energies are in kcal/mol.
